# Integrated species–phenon trees: visualizing infraspecific diversity within lineages

**DOI:** 10.1038/s41598-019-55435-w

**Published:** 2019-12-12

**Authors:** Abdullah Khan Zehady, Barry G. Fordham, James G. Ogg

**Affiliations:** 1 Department of Earth, Atmospheric, and Planetary Sciences, Purdue University, West Lafayette, IN USA; 2 Research School of Earth Sciences, Australian National University, Canberra, ACT Australia; 3 State Key Laboratory of Oil and Gas Reservoir Geology and Exploitation, Chengdu University of Technology, Chengdu, Sichuan China

**Keywords:** Phylogeny, Speciation

## Abstract

The unprecedented detail with which contemporary molecular phylogenetics are visualizing infraspecific relationships within living species and species complexes cannot as yet be reliably extended into deep time. Yet paleontological systematics has routinely dealt in (mainly) morphotaxa envisaged in various ways to have been components of past species lineages. Bridging these perspectives can only enrich both. We present a visualization tool that digitally depicts infraspecific diversity within species through deep time. Our integrated species–phenon tree merges ancestor–descendant trees for fossil morphotaxa (phena) into reconstructed phylogenies of lineages (species) by expanding the latter into “species boxes” and placing the phenon trees inside. A key programming strategy to overcome the lack of a simple overall parent–child hierarchy in the integrated tree has been the progressive population of a species–phenon relationship map which then provides the graphical footprint for the overarching species boxes. Our initial case has been limited to planktonic foraminfera via Aze & others’ important macroevolutionary dataset. The tool could potentially be appropriated for other organisms, to detail other kinds of infraspecific granularity within lineages, or more generally to visualize two nested but loosely coupled trees.

## Introduction

Recent advances in molecular systematics are not only building a tree of life but are also allowing the evolutionary structure within a species to be investigated more finely than ever. It is now quite commonplace for species to be revealed as a rich array of clades at multiple taxonomic levels, of difficult-to-assign infraspecific groupings, of cryptic or pseudocryptic species, or of variously fragmented haplotype-phylogeographic populations (follow-up statements in later sections will provide references).

Less well known is that in paleontology an analogous morphotaxonomic granularity within species lineages, long expressed as formal or informal infraspecific morphotypes and the like, is now being portrayed phylogenetically and on a large taxonomic scale — at least in one fossil group. This group is the Cenozoic macroperforate planktonic foraminifera: Aze & others (2011)^1^ have proposed for it a species-lineage evolutionary tree comprising 210 lineages, each explicitly incorporating (usually multiple) component morphotaxa.

The macroevolutionary properties of Aze & others’ lineage phylogeny have attracted considerable interest (e.g.^2^). However, this is not so when it comes to the phylogeny’s microevolutionary ramifications, which in fact do include a granularity within their species lineages implied by the morphotaxa. We contend that this microevolutionary aspect of Aze & others’ phylogeny also deserves attention by evolutionary researchers, especially as most of those species lineages which have survived to the present have now been subjected to molecular analyses, placing planktonic foraminifera in quite a unique position to compare in detail infraspecific diversity from both living and fossil perspectives.

One could nominate important reasons why the focus on Aze & others’ phylogeny has been macro- rather than micro-evolutionary. However, we suggest that there has also been a merely practical hindrance to looking into the content within these lineages — Aze & others’ visuals were simply too complicated to employ for this purpose. This is because they provided the morphotaxonomic content for their species lineages indirectly via a separate evolutionary tree for their morphotaxa, and because the internal timing and the topology of both lineage and morphotaxic trees do not match (deliberately so, but more on that later). So, for the reader to appreciate that content, considerable dexterity is required when comparing corresponding parts of each tree: visual dexterity for their tree figures, arithmetic dexterity for their spreadsheet listings (which date and decode their lineages with regard to morphotaxa).

Given the special research potential provided by planktonic foraminifera, we seek to remedy the brain teaser presented by the trees of Aze & others by providing a visualization tool which combines both into a single graphic. In fact, as we will demonstrate, not only does the new integrated tree capture all relevant aspects of each component tree, its additional visual aids delineate when and from where morphological changes are implied to arise both within the species lineage and across proposed speciation events. So it encourages a critical appraisal of the dynamics of morphological evolution in relation to speciation (cladogenesis) and lineage continuity (anagenesis).

Below, we briefly introduce the case study of Aze & others (2011) and we explain why their terminology needs to be adjusted for our tool to bridge both paleontological and living research practices in planktonic foraminifera. We then show that the tool is needed in order to merge two trees (of species lineages and of phena) that are decoupled from each other. In § Results the tool is introduced and applied to the case study, followed by a discussion highlighting mutual learnings it can help broker between paleontological and living research perspectives, as well as other potential applications. § Methods deals with the programming behind the tool and its challenges. But firstly, below we seek to briefly contextualise where visualization within species is at.

## Visualizing Infraspecific Relationships

Graphically depicting evolutionary relationships within species—and specifically between entities or taxa below the species level — has been on the agenda even since Darwin. Darwin did so with his tree-like diagram in the *Origin of Species*^3^ (between pp. 116 and 117) where he envisaged lineal continuity between forms, varieties, subspecies, and species^4^. However, in our contemporary evolutionary lexicon of species as metapopulation lineages^5^, one might presume much of infraspecific usage to be outdated, and so visualizing them to be largely superfluous. That is, until you look a little further. Depictions of varieties and subspecies, for instance, are not hard to find within state-of-the-art taxonomic studies drawn from across the organic world — be they about pathogenic bacteria^6^, reed grasses^7^, endangered crayfish^8^, or titi monkeys^9^. One could in fact get the impression that even the phenon has an assured place in current molecular microbial studies^10^.

A key incentive in recent times to visualize relationships within species has been the discrimination increasingly afforded by molecular systematics. Earlier, in both established systematic traditions as well as initial molecular-systematic studies, the focus was and continues to be on superspecific relationships: from specialist research within, say, a family to collaborative ventures aimed at the tree of life. For this context, species are inputs to the study (as such, or as representatives of higher taxa) and their validity is a given. However, the subsequent blossoming of molecular-phylogenetic studies has for the first time allowed in-depth examination of evolution from the gene up. A key component of this research centres around and below the species, with much of the focus in case studies now reframed as the “species complex”^11–19^. And exciting challenges now involve rethinking of once-given species, including their genetic discrimination^20^ as well as delineation of their putative evolutionary components.

In this contemporary research environment, a variety of approaches to recognizing and visualizing evolutionary entities around and within the species are being tried. Often, gene trees for species complexes or, say, closely related genera are fully subdivided into clades recognized as species — though the choice and extent of clades is usually manual and pragmatic, influenced by prior systematics^12^, and the genetic complexity within and among prior species may raise the level needed to recognize reliable groupings to, say, nominal subgenera^11^. In other cases, for example: molecular support for prior species may prove poor^13^; geographic variants may cut across prior species^15^; or a formal–informal hierarchy of genera, infrageneric clades, species, and unlabeled infraspecific groupings may be viable for most prior species, but there may also be a desiderata of cryptic and pseudocryptic species and species with ambiguous genetic distances^16^. For the geographic–genetic differentiation of populations within species or species complexes gained from haplotype phylogeography, mitochondrial-DNA trees are often complemented by annotated networks and ordination of haplogroups^21–24^. Given the many considerations needed to evaluate gene trees, enhanced visualization approaches have been developed, including embedded display of database information, gene sequences, and phylogeographic maps^25,26^.

Another layer of interpretative and visualization complexity comes from addressing incongruence between gene and species trees arising from incomplete lineage sorting (deep coalescence), horizontal gene transfer, or gene duplication or loss/extinction events^27–29^. This applies in particular to very recent speciations or, more generally throughout deep time, when successive speciations are rapid relative to effective population sizes^30^. So this is especially pertinent for visualizing genetic dynamics within the species, particularly when associated with speciation, potential or eventual. Computational inference of species trees from gene trees^31^ can now employ a rapidly growing array of methods^32^. When applied to multigene studies, the challenge of visualizing many gene trees^11,17^ may be best guided by the image of a “cloudigram”^29^ rather than simple lineal trees and, apart from networks and ordination^14^, visualizations such as rotatable three-dimensional trees^33^ and tanglegrams^34^ become relevant.

Despite the sophistication which molecular phylogenetics can now bring to differentiation within the species, translating that into phylogenies against deep time has almost always been confined to a simple, and so usually simplistic, projection employing fossil or paleogeographic evidence to date and calibrate selected nodes^35,36^. So the deep-time history of the molecularly detected infraspecific entities remains largely illusory. Studies which do attempt to detail this history — on groups considered to have an instructive fossil record^37,38^ — will usually need to raise their taxonomic focus for deep time to that of the encompassing species or species groups, providing only a qualitative context to their enhanced depiction of living infraspecific diversity.

## The opportunity now afforded by planktonic foraminifera

A taxonomic area which does offer hope for detailing evolution of molecularly or otherwise delineated entities within species through deep time is that of microfossils^39^. These phylogenetically diverse groups are united by typically highly rich fossil records from which abundant assemblages can be recovered from tiny samples. Where their fossilisable microscopic parts appear to preserve evolutionary change along stratigraphic sequences, biostratigraphers have typically captured those changes taxonomically, often using a variety of infraspecific labels, formal or informal^40^. So for those microfossil groups that include living representatives, contemporary molecular studies are now providing the potential to compare living infraspecific diversity with that implied through deep time.

A notable example of this opportunity is given by foraminifera, especially the fifty or so living species that are planktonic^41^. A growing body of molecular studies on planktonic foraminifera are not merely applying stratigraphic evidence to calibrate their genetic trees against geologic time, but are explicitly exploring a deep-time context for their molecularly detected infraspecific entities^42–52^. And added to this, their biostratigrapher colleagues have quite recently provided a key macroevolutionary framework for infraspecific diversity through the Cenozoic. For the largest living group of planktonic foraminifera, the macroperforates, Aze & others^1^ have proposed phylogenies not just of the usual biostratigraphic taxa but also one which integrates these taxa within whole-species paleobiological lineages, and so depicting deep-time polytypic species directly comparable to living species.

It is Aze & others’ conceptualisation which we consider adds an exciting input into the interchange between living and fossil research, and which forms the case study for our visualization tool. But before introducing the tool, we need to explain why we have avoided their term, morphospecies, and then explain why the tool is needed.

### Avoiding “morphospecies”

**Figure 1:**
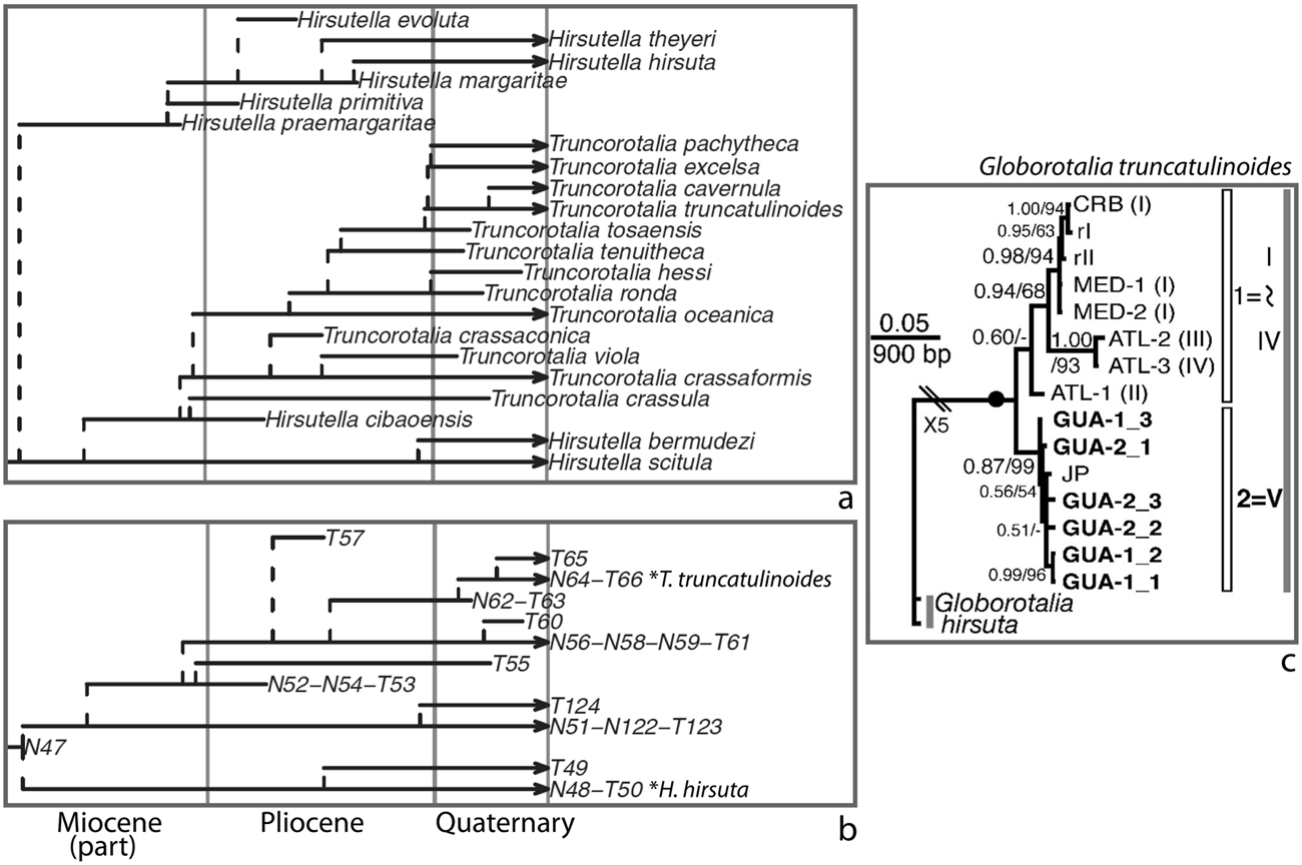
Paleontological and molecular approaches to infraspecific terminology: planktonic foraminifer *Globorotalia*/*Truncorotalia truncatulinoides*. (**a,b**) A paleontological approach. Portions (after Fig. 5E of^1^) of trees of morphospecies [evolutionarily overlapping morphotaxa] (**a**) and lineages [paleobiological species] (**b**): living Lineage N64-T66 (*T. truncatulinoides in b) includes three living morphospecies (in a), *Truncorotalia truncatulinoides*, *T. excelsa*, and *T. pachytheca* (*T. cavernula* originated in this lineage, but budded into its own living Lineage T65); for comparison with the outgroup in (**c**), these portions of the trees embrace living Lineage N48-T50 (*H. hirsuta in **b**) which includes living morphospecies *Hirsutella hirsuta*; arrows indicate still living. (**c**) A molecular approach. Consensus molecular tree for the morphospecies [cryptic-species complex] *Globorotalia truncatulinoides*, using morphospecies *G. hirsuta* as the outgroup (after Fig. 4b of^57^): two major clades 1 (“1 = I ~ IV”) and 2 (“2 = V”) comprising five genotypes [cryptic species] I – IV and V, respectively. Note: *Globorotalia* versus *Truncorotalia*, *Hirsutella*, etc., are alternative nominal genera, the former preferred for molecular workers’ broader less-settled sweep across living species, the latter for those paleontologists keen to emphasise temporally deep but taxonomically confined lines of descent.

**Figure 2:**
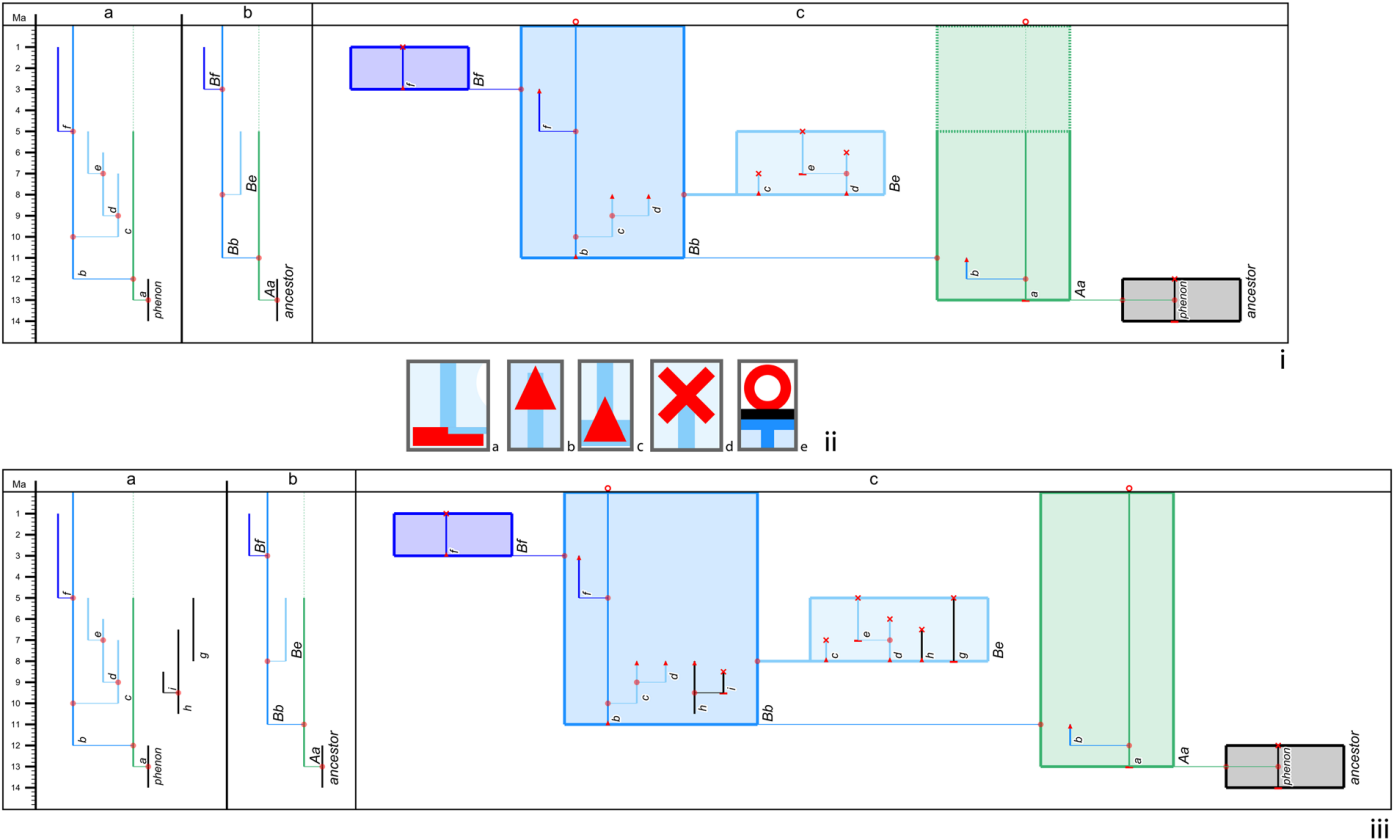
Sample derivation of an integrated species–phenon tree. (i) Improvised example of evolutionary trees against geologic time scale (Ma), depicting a “black” ancestor giving rise to a “green” descendant, in turn to a “blue” group (“medium blue”, ancestral to “light blue” and “dark blue” descendants); all range lines effected in the same line style (“frequent”), except for the upper/later portion of phenon *a* and species *Aa* (“conjectured”; for *TimeScale Creator* line styles, see pp. 48–49 of^81^); drawn by *TimeScale Creator* datapacks (§ Data availability). Column a = phenon tree. Column b = species tree. Column c = integrated species–phenon tree. (ii) Enlarged view of guide symbols attached to phenon range lines in column c to provide stratigraphic or phylogenetic context, including their parts broken between species boxes: (**a**) range origin; **(b**) top of broken part of range, to transfer to the next descendant species box; (**c**) bottom of broken part of range, to transfer from the immediate ancestral species box; (**d**) range extinction; (**e**) still living. (iii) Incorporation of disconnected phenon trees and ranges (see § Methods); evolutionary-tree series of (**i**), with added phenon range and phenon tree in black. Column a: the original coloured phenon tree, additional phenon tree (**h–i**), and additional phenon range (**g**), all displayed in the same column, from left to right in order of First Occurrence (the default option). Column b: as in (i); the additional black phena, (**g–i**), from column a have been assigned to the *Bb*–*Be* lineage series, but their ancestor–descendant relationships with the original coloured phena are considered poorly known. Column c: integrated species–phenon tree, with the disconnected phenon tree (**h–i**) and range (**g**) positioned in their corresponding species boxes.

As already alluded to, Aze & others^1^ presented two parallel evolutionary trees for Cenozoic macroperforate planktonic foraminifera: one of biostratigraphic taxa, traditional for micropaleontology, accorded *Genus species* binomina, and termed morphospecies; and another of newly introduced biological-species lineages constructed of mostly multiple morphospecies and given codes. These morphospecies are not chronospecies, they do not subdivide lineages into temporal chunks but rather into segments in morphological space (see p. 195, Text-Fig. 2 in^53^; pp. 903–905, Fig. 1b in^1^; pp. 6–7 in^54^). Despite the pragmatic role that this kind of morphospecies plays in paleontology, in the context of informing and interacting with research into living planktonic foraminifera and its conventions, this usage of “morphospecies” becomes problematic, as seen in the following example.

Aze & others portrayed the later phylogeny of the well-studied living *Truncorotalia* (or *Globorotalia*) *truncatulinoides* as two successive Pliocene–Quaternary lineages comprising six morphospecies (Fig. 1a,b; Lineages N62-T63 and N64-T66; morphospecies series beginning with *T. tenuitheca*). As a result, the living *T. truncatulinoides* comprised one lineage but three morphospecies, *T. truncatulinoides, T. excelsa*, and *T. pachytheca*. On the other hand, molecular researchers^43,45,55–59^ have consistently considered living *T*./*G. truncatulinoides* a single “morphological species”. This characterisation references an earlier taxonomic tradition which actually included most micropaleontologists whereby only one nominal species, *T*./*G. truncatulinoides*, was recognised for the entire Quaternary lineage (and so also for the present day), whether infraspecific taxa were also delineated^60–64^, or not^65–69^.

This disjunct between the terminology of Aze & others and molecular researchers has been further underlined by the latter’s practice of using “morphospecies” as a shorthand for their “morphological species”. So, in current parlance, the living *T*./*G. truncatulinoides* contains three morphospecies in one part of the literature but one morphospecies in another. It is somewhat ironical then that the above-quoted molecular research on *T*./*G. truncatulinoides* has delineated five genotypes, possibly indicative of two genetic lineages, one with four putative species (Fig. 1c). And, though the relationship between these genotypes and the three morphospecies recognised by Aze & others is apparently yet to be addressed, the potential for a coming together of paleontological and molecular concepts for a one-to-one genotype–morphospecies correspondence for these taxa appears poor. Rather, the apparently exclusively subtropical extent (Caribbean, Mediterranean and Canary Islands, west Pacific)^60,61^ of the two ancillary morphospecies, *T. excelsa* and *T. pachytheca*, suggests both might be subsumed within only a single genotype which would also include the nominate *T*. *truncatulinoides* (see the distribution of genotype Type II in Fig. 7 of^56^). So, without much more investigation, these three paleontological morphospecies seem unlikely to contribute much to a deep-time context for the five genotypes.

**Figure 3:**
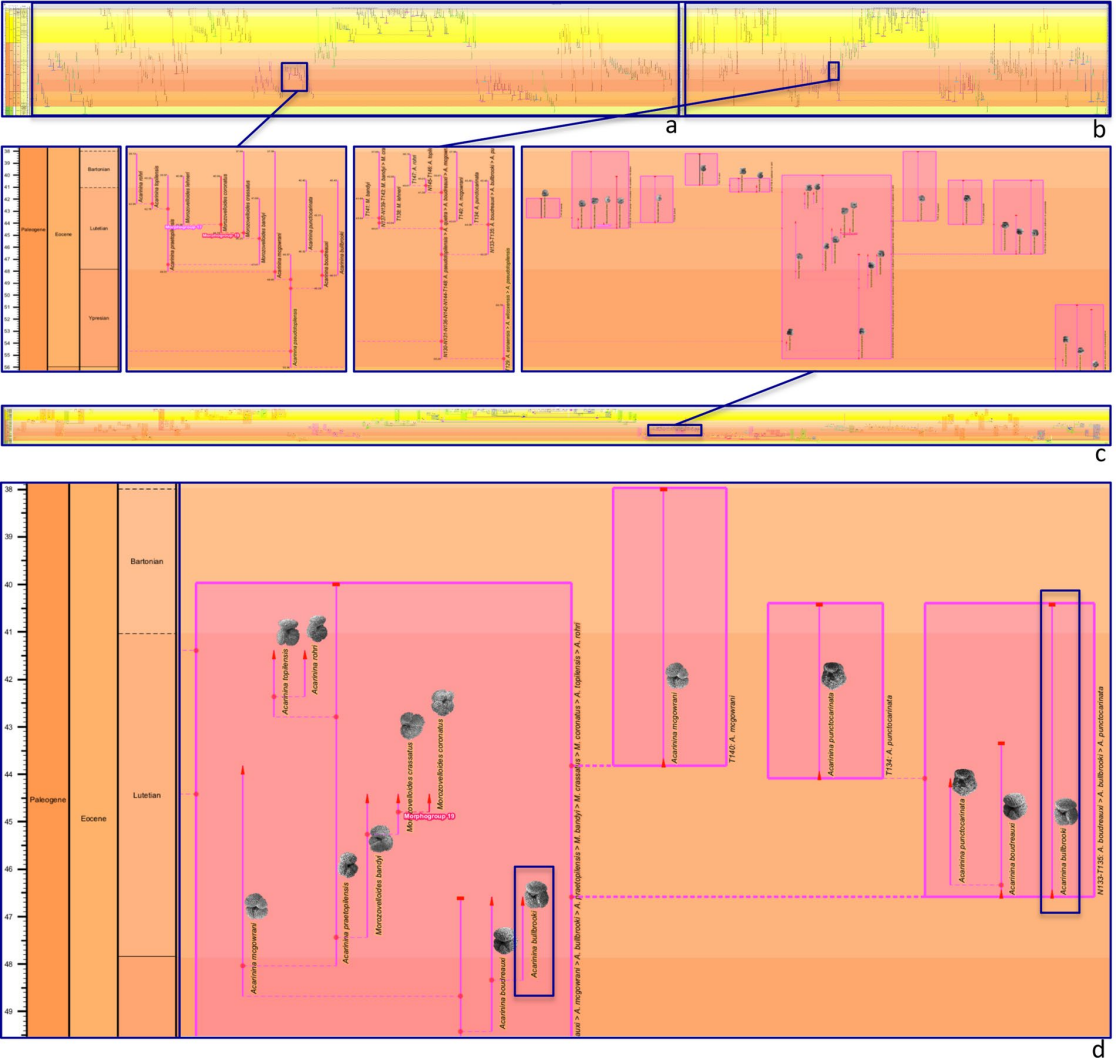
Derivation of an integrated species–phenon tree for the case study of Cenozoic macroperforate planktonic foraminifera^1^. Evolutionary-tree charts drawn by *TimeScale Creator* datapacks, (**a–c**) against entire Cenozoic time scale (the last 66 Myr), with insets of a Ypresian–Bartonian (Eocene) clade which begins with a phenon (morphospecies *Acarinina pseudotopilensis*, **a**), or with its corresponding species (Lineage N130-N131-N136-N142-N144-T148*, **b,c**); clade shown within Fig. 5C of^1^ and Figs. 7f and 20 of ^54^; tree colours and groupings by morphogroup; background coloured by stages (“Chronostrat” option). (**a,b**) Entire phenon (morphospecies) and species (lineage) trees^54^, respectively, each with inset (below). (**c**) Entire integrated species–phenon tree, with inset (above); this tree introduced herein (§ Data availability); images^85^ for morphospecies added as heuristics to help appreciate the break-up of the phenon tree within the species tree, but these images are not authoritative. (**d**) Detail (upper right) from inset of (**c**); red range guides on phenon range lines as in Fig. 2ii; time interval is late Ypresian–Bartonian (Eocene). Note the breaking graphically of the range line of the highlighted phenon (morphospecies *Acarinina bullbrooki*) between the ancestral species box (Lineage N130-N131-N136-N142-N144-T148, far left) and its descendant species box (Lineage N133-T135, far right). *With regard to species-lineage labels in (**c,d**), note that, in order to make it easier to follow the lineage codes of Aze & others, as part of the transfer to *TimeScale Creator*^54^ these labels were programmatically appended with a list of included phena. This label then becomes “N130-N131-N136-N142-N144-T148: A. pseudotopilensis > A. quetra > A. boudreauxi > A. mcgowrani > A. bullbrooki > A. praetopilensis > M. bandyi > M. crassatus > M. coronatus > A. topilensis > A. rohri”: this lineage happens to be one of their more inclusive!

Examples other than *T*./*G. truncatulinoides* would point to similar problems with the “morphospecies” but for different reasons. This is because in molecular studies of living planktonic foraminifera the relationship between the nominal species, usually labeled “morphospecies”, and genetic species is a complicated one^56,70^: about half of the nominal species analysed each have a one-to-one relationship between nominal and genetic species (though high genetic diversity can nonetheless occur at low genetic levels, as in *Pulleniatina obliquiloculata*^70^); in another half of the nominal species, each contain multiple genotypes suggestive of multiple putative species or, in fact, genetic lineages; the reverse situation of multiple nominal species containing a single genotype is rarer but represented by *Trilobatus*, (Fig. 3 in^70^) apart from the somewhat contrived case of *T*./*G. truncatulinoides*; and finally in some cases a nominal–genetic hierarchy (in either direction) may break down because multiple nominal species are phylogenetically interwoven between multiple genotypes (as in *Globigerinella* and *Globigerinoides ruber* sensu lato^70^).

This discussion suffices to demonstrate the ambiguity and ineffectiveness of “morphospecies” as a term applied to planktonic foraminifera when wanting to integrate current living and paleontological research. Seeking an alternative taxonomic category, the only other option in zoology would be the subspecies, but it would suffer analogous inconsistencies, especially given its important conceptual role in evolutionary practice. So, in applying the case study of Aze & others to our visualization tool, we have retreated to informal practice and replaced Aze & others’ “morphospecies” with Mayr’s use of phena^71,72^, as applied previously to biostratigraphic taxa in planktonic foraminifer^63^, more generally^73,74^, and of course to prokaryotes^75^. This informal paleontological taxonomy then mirrors the emerging informal, though rules-based, system of molecular nomenclature intended to parallel and link to formal nomenclature for planktonic foraminifera and other organisms^70^. And for Aze & others’ other tree, we simply refer to their biological-species lineages as species. Overall, these two adjustments to the terminology employed by Aze & others allow us avoid the very confusing “morphospecies” but otherwise run with the emerging language of molecular research, and comfortably discuss species in an uncontroversial and broadly applicable way.

### Why our visualization tool is needed

The above-discussed developments in the study of planktonic foraminifera, from both the living and fossil research communities, can provide a much clearer rationale for extending the living infraspecific diversity of planktonic foraminifera into deep time. There is, however, a practical issue in applying Aze & others’ phylogenies to this purpose. Their separate trees of species (biological-species lineages) and phena (biostratigraphic “morphospecies”) have topologies which do not correspond in a straightforward way. This is because the timing they interpreted for the emergence of species in deep time, though influenced by the stratigraphic ranges of related phena, was based on different evidence — the tracing of morphological clusters of collections of specimens (not taxa, formal or informal) along stratigraphic sequences (p. 195, Text-Fig. 3 in^53^; Fig. 2 in^1^; § Ancestor–descendant relationships in^54^). This methodology disarticulates the origins of species from those of phena, and so allows phenon ranges to pass unbroken from an ancestral species to one of its descendant species (see morphospecies m3 in Fig. 1b of^1^; note also in Fig. 1a,b herein that, for instance, phenon *T. truncatulinoides* appears in the uppermost Pliocene so, when Lineage N64-T66 buds of from its ancestral Lineage N62-T63 in the early Quaternary, that phenon would carry over from its membership of the latter to that of the former). This breakdown in the simple species–phenon hierarchy also underlines the inappropriateness of employing, for example, nominal subspecies for these biostratigraphic taxa.

Making sense of these biostratigraphers’ out-of-synch evolutionary trees of species and phena turns out to be a visual brain-teaser. In this paper our visualization tool provides a graphical solution to this. Our integrated species–phenon tree takes the species tree and widens each species range line into a “species box” within which is displayed that species’ algorithmically determined portion of the tree of phena. Within a species box, symbols attached to the range lines of the phenon tree provide guides to both the origins of the phenon phylogeny from ancestral species and its continuance into descendant species. And all this is accomplished programmatically from a single extra data link between the two trees’ datasets.

We now describe the integrated species–phenon tree, apply it to this case example of Cenozoic macroperforate planktonic foraminifera, and then suggest how these trees might enhance broader research into infraspecific diversity, especially that which we seek to detail through deep time.

## Results

### The software tool

Our integrated species–phenon tree, part of the *TimeScale Creator* software package (§ Methods), is a species tree drawn as a SVG chart against geologic time but with the species expanded into boxes in which is projected an underlying infraspecific (phenon) tree (Fig. 2i). Those infraspecifics could be taxa (subspecies, botanical varieties or forms, prokaryotic infrasubspecifics, etc.) or other entities of interest (molecular OTUs, gene sequences/loci, biostratigraphic taxa, etc.).

The key incentive for a software tool to bring together both species and phenon trees is that, though the trees refer to the same organisms, they have different topologies, in terms of both the nature and number of entities recognized and the timing of their evolution. Even in our simple improvised example of just a few taxa (Fig. 2i), observing which species (column b of Fig. 2i) contain which parts of the phenon tree (column a) is not easy. So our tool does that for you (column c). It also provides range guides (Fig. 2ii) to better appreciate the context of the now broken-up parts of the phenon tree; these make clear the full durations of phena and easily distinguish these from transfers of the phena between species. So now the integrated tree can provide not only all the information contained in the individual species and phenon trees, but also enhance understanding of the morphologic/genetic/etc. variability within species, as well as the timing of evolutionary change within and between species, including what morphological change taxa capture within and across speciation events. In so doing it encourages an examination of the dynamics of morphological evolution in relation to speciation (cladogenesis) and lineage continuity (anagenesis).

### Case example

The practical viability of our tool finds a ready check in Aze & others’^1^ jointly published species and phenon trees for Cenozoic macroperforate planktonic foraminifera, especially apposite as they were recently transferred onto the *TimeScale Creator* platform^54^. As already alluded to above (§ Avoiding “morphospecies”), their trees constitute a major dataset of large and complex trees comprising 210 species (their biological-species lineages) and 339 phena (their “morphospecies”) over a time interval of 66 Myr. To systematically recognize, let alone name, species lineages is unusual in micropaleontology; Aze & others introduced codes for their lineages, e.g., N133-T135, by concatenating numerical codes given to their included lineage-tree leaves, prefixed with N if internodal, T if terminal. Their “morphospecies”, given as *Genus species* binomina, followed a convention in their field to employ distinctive, especially biostratigraphically useful, morphologies as formal taxa. Moves are underway to revise the trees of Aze & others to incorporate the recently published major changes to Oligocene taxonomy and phylogeny^76^ and analogous but longer-term updates expected from a fledgling Neogene Planktonic Foraminifera Working Group.

Within Aze & others’ trees the rate and content of macroevolutionary change in terms of species and infraspecific taxa is highly variable (Fig. 7 in^54^). The relationship between species and phenon trees, constructed stratophenetically (Fig. 2 in^1^), is similarly variable: for example, though origins for about 40% of the species lineages coincide with one of their contained phena (see “morphospecies” in pdf p. 18 in^54^), many phena originate within species lineages and a substantial number cross from ancestral to descendant lineages. So envisaging, for example, the phenon content of species lineages from the trees figured in the original paper is just too difficult for the average observer (compare in^1^ the top and bottom parts of Fig. 5A–J, or the separately drawn “morphospecies” and lineage trees in Appendices S2 and S3). And this visual challenge is only partially alleviated by enhanced displays (§ Linkages between morphospecies and lineage trees in^54^) introduced to assist with this.

Applying the new integrated species–phenon tree to bring these trees together results in an even wider tree (Fig. 3c); nonetheless the new chart still draws quickly, a testament to the scalability built into the Java code of *TimeScale Creator* software^77^. Closer inspection demonstrates that the phenon range lines (Fig. 3a, inset) have indeed separated at species lineage origins (Fig. 3b, inset) to transfer into their respective species boxes (Fig. 3c, inset). The much easier access to species–phenon relationships afforded by the integrated tree is underlined by comparison of these species boxes with our earlier attempt (Fig. 7f in^54^) at a similar effect by manually overlaying lineage outlines onto the phenon (“morphospecies”) tree. The integrated tree also features range guides (Fig. 3d; legend in Fig. 2ii) to help the viewer appreciate the stratigraphic and phylogenetic context for any phenon range, and especially to follow the trajectory of those that cross species boxes (note the phenon highlighted). In addition, as already stated, the integrated tree brings with it all the information from the individual species and phenon trees. Viewed interactively on the *TimeScale Creator* platform, this includes the mouse-over pop-ups which, in this case example, include a wealth of taxonomic, morphologic, ecologic, and biostratigraphic detail from the back-end database (including the lineage–morphospecies linkages already mentioned)^54^.

## Discussion

**Figure 4:**
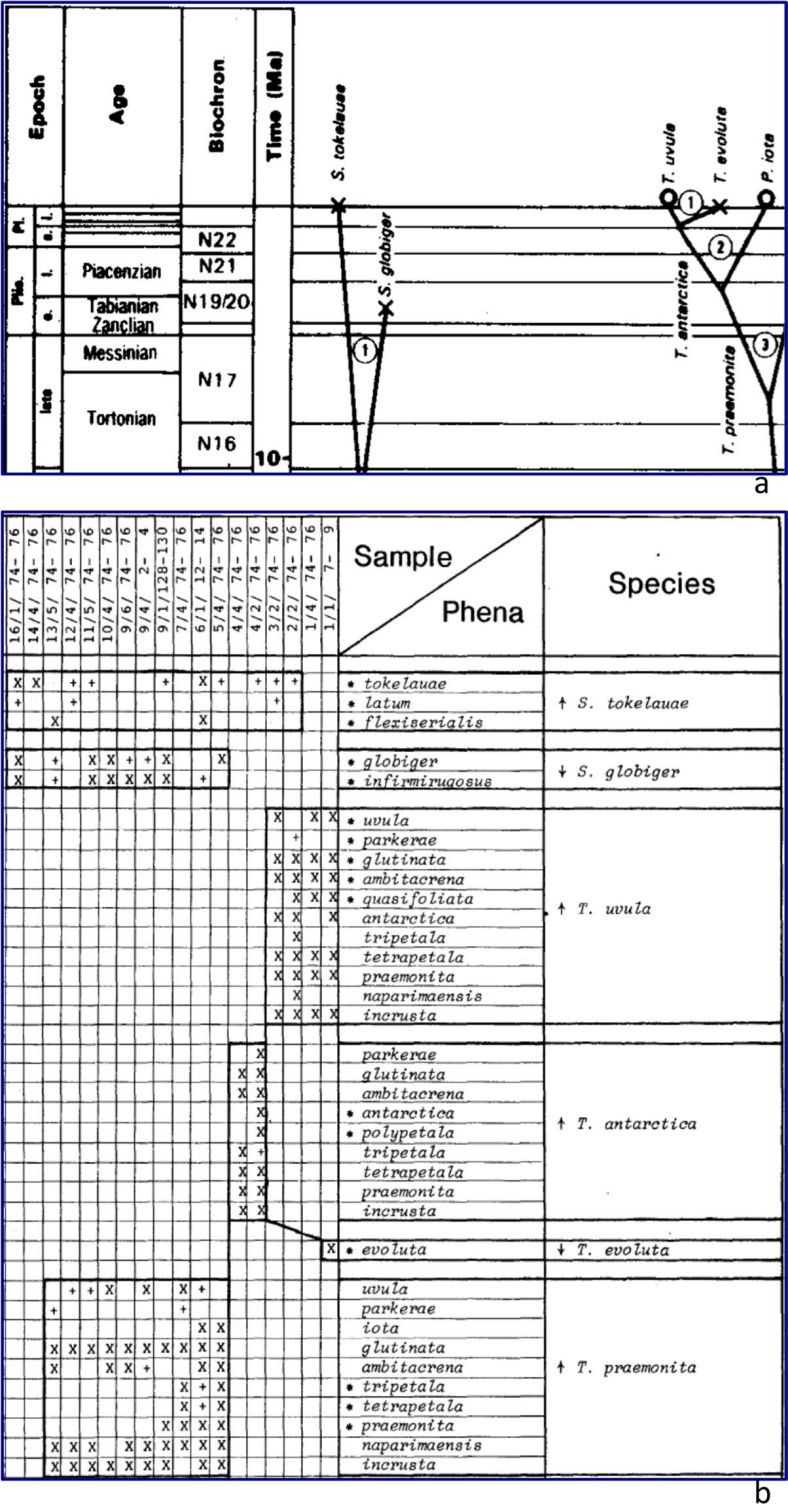
Earlier thinking towards species–phenon trees. A small portion of the phylogeny and stratigraphic charts from a study^63,86^ of Cenozoic planktonic foraminifera: a window limited to the last 10 Myr, focusing in on *Streptochilus* and *Tinophodella praemonita* and descendants (see original for details). (**a**) Portion of species tree (Text-Fig. 2b of ^63^, upper-left). (**b**) Corresponding stratigraphic-distribution chart for Deep Sea Drilling Site 208 (Table 3a of^63^, portion): species boxes arranged and linked according to the species tree, with occurrences of included phena listed by stratigraphic order of primary types.

We have already alluded to the many approaches to visualizing infraspecific content of species through time, so our integrated species–phenon tree has a rich heritage. It could, for instance, be traced back to the then newly enthused evolutionary paleontologists of the late nineteenth century^78^. More specifically, our formulation was anticipated by predigital species–phenon boxes employed to document stratigraphic distributions (Fig. 4b) within the context of a species tree (Fig. 4a). Also, within the context of documenting infraspecific content, there is the “phenon group”, an informal category uniting homologous phena in multielement fossils^79,80^. Interestingly, if trees of phenon groups were constructed, given that both descendants at speciation would often retain the same phena for some phenon groups, the relationship of phenon-group trees with their species trees should bear similarities to relationships between gene trees and their species trees — bringing us back to some topics we raised above.

**Figure 5:**
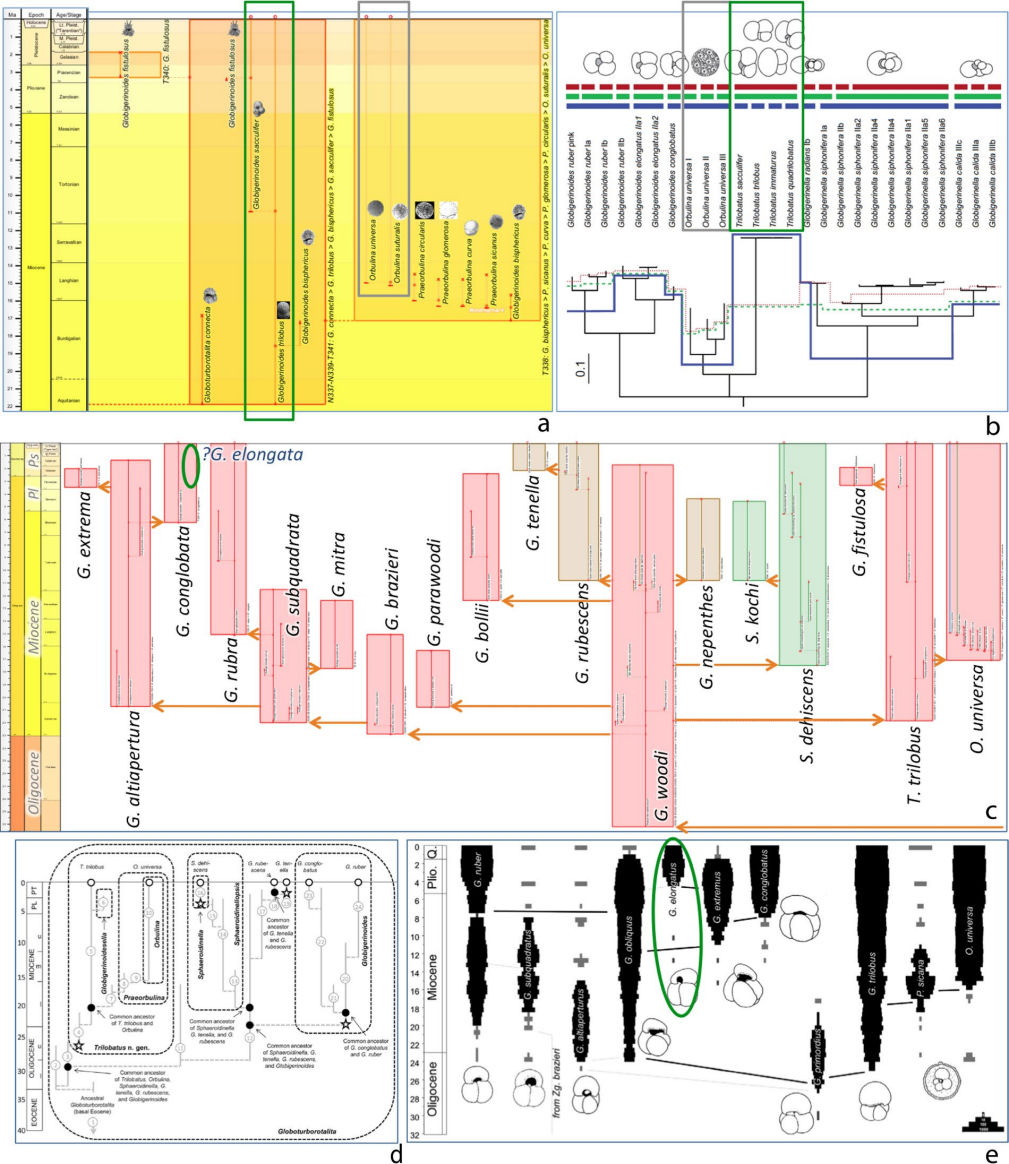
Mutual learnings between integrated species–phenon trees and molecular trees. (**a,b**) Living species of planktonic-foraminifer genera, *Orbulina* and *Trilobatus*, and their evolution during the Neogene. (**a**) Portion with these two genera from the integrated species–phenon tree of the case study of Cenozoic macroperforate planktonic foraminifera (see Fig. 3c). (**b**) A molecular phylogeny of those two genera and related groups (Fig. 3 of ^70^). *Orbulina* (grey boxes): rather than a single species lineage (T338) with two phena that are still living, the genetics point to the need for stratophenetic studies to check for progressive speciation through the Miocene–Pleistocene which resulted in three living species. *Trilobatus* (green boxes): stratophenetic studies have already placed the four genetically almost identical nominal species employed by molecular studies into a single species lineage (N337-N339-T341; with two of those nominal species portrayed as intergradational phena, two synonymised). (**c–e**) Phylogenies since the late Oligocene of planktonic foraminifera related to *Globigerinoides*. (**c**) An annotated portion from the integrated species–phenon tree of the case study of Cenozoic macroperforate planktonic foraminifera (see Fig. 3c); coloured by ecogroups; species binomina are not authoritative (genus epithets after^87^); details of phena within species, etc. are viewable interactively when the datapack is loaded onto the *TimeScale Creator* platform (§Data availability). (**d**) A stratophenetic scheme influenced by molecular phylogenetics (Fig. 5 of ^87^; see for details). (**e**) A molecular–stratigraphic scheme (Fig. 6 of^49^; see for details). The molecular study (**e**) suggested species *G. elongata* (green ellipse) is a living descendant of *G. conglobate* and as-yet unrecognized by the stratophenetic approaches [see green ellipse in (**c**)] as it is a homeomorph of *G. rubra* (though this would be a relatively minor convergence as both share quite a close common ancestor, *G. subquadrata*).

Coming forward to contemporary research related to our case example of planktonic foraminifera, parallels can be made, especially with regard to living representatives, between the perspective the integrated tree brings and that coming from molecular studies. For instance, the integrated tree explicitly visualizes species as dynamically evolving polytypic lineages, presenting them in terms of a two-level species–phenon hierarchy (Fig. 5a). Meanwhile, some molecular studies are not only genetically circumscribing nominal species and separating out misunderstood cryptic look-a-likes, they are also laying the basis for an informal (but rules-based) multilayered infraspecific taxonomy: a visualization that recognizes the complexity of species lineages and their phylo-geography/ecology/oceanography in terms of genetic lineages, putative species, and populations^70^ (Fig. 5b). Given that deep-time (for example paleoceanographic) applications of planktonic foraminfera depend on developing analogous levels of understanding, one wonders if the species–phenon depiction will continue to be adequate into the future, in regards to either its mere two-level hierarchy or the way phena (as species-group taxa) are currently employed. However, compared to other phylogenetic schemes currently employed for deep time, the species–phenon tree does provide both a comprehensive and specific set of proposals and timings, in terms of both species and phena (Fig. 5c). And some of these could potentially be amenable to, for example, molecular dating and phylogenetics. And, despite the rich fossil record of planktonic foraminifera, taking the results of molecular phylogenetics into deep time can usefully suggest parts of this record needing reexamination for stratophenetic phylogenetics (Fig. 5d,e: the case of *Globigerinoides elongata*).

Beyond Aze & others’ phylogeny of planktonic foraminifera, our integrated tree should have immediate application for similar cases where the fossil record is rich enough to motivate the proposal of phylogenetic trees of not just fossil morphotaxa but paleobiologic species. And, if those organisms have living representatives, a similarly fruitful interchange with molecular and other living research would be expected. But we would also encourage researchers with other kinds of temporal infraspecific information to consider placing that within the expandable graphic provided by our tool in order to visualize the phylogenetic context and detail of that information. This information need not be in tree form. For instance, the graphic could be used to display, say, the temporal positions and proposed species memberships of specimens, especially when at the working stage of a phylogenetic study. More generally in a more purely graphical context, a visualization tool which can bring together two nested trees that can nonetheless remain loosely coupled may have broader application.

## Methods

### Introduction and rationale

Our integrated species–phenon tree takes a species tree (Fig. 2i, column b) and phenon tree (column a) for the same organisms, both set against geologic time, and combines the two (column c). This is accomplished by allowing the species time ranges (leaves) of the species tree to expand laterally into rectangles (“boxes”), breaking up the ranges of the phenon tree at the branch points of the species containing them, and then placing the broken-up parts of the phenon tree into their corresponding species boxes. Guide symbols (Fig. 2ii) are added to the extremities of each of the broken phenon ranges so as to indicate their context (their origin, their extinction or still-living presence, or transfer from their ancestral species or to their descendant species).

The new integrated tree is drawn within the Java-based *TimeScale Creator* (*TSCreator*, *TSC*) visualization software package, as were its component species and phenon trees. The integration is achieved with minimal additional data input — a single extra entry for each phenon, denoting the species in which its range ends — the tool does the rest. This minimal indication of links between the two component trees avoids the introduction of errors from manual tree compilation, likely with even the most meticulous effort when the dataset is large (e.g., for our case example^1^, the original listings in Appendix S5 of the 339 morphospecies in the 210 lineages contained a small % of such errors, understandably).

Visualization of the integrated tree on the same platform as its component trees makes it easy to transfer all of the latters’ graphical features as well: line styles, colours, thumbnail images, pop-ups, and, most importantly, the exact dimensions of the tree topologies. An additional flexibility built into the integrated-tree tool allows the inclusion of phenon ranges within species boxes for any phena not connected into the original phenon tree. Similarly, any phenon trees/subtrees disconnected from the main phenon tree will also be drawn within their species boxes; this could be employed for depiction of additional infraspecific categories. A further feature allows the user to provide alternative labels for phena; this could be employed, for example, to label phena with only a single species epithet (*species*, of *Genus species*) which is then easily visually distinguished from the binomen of its enveloping species (*Genus species*).

Programming the new integrated tree presented significant challenges. The key issue for the previously coded evolutionary tree for *TimeScale Creator* concerned collation and coordination of widths of diverse graphical components, quite a task in itself. The integrated tree adds further complications arising from the lack of a simple overall parent–child hierarchy, with the result that, for example, the number of breaks within phenon ranges is not easily determined up-front, requiring greater preemptive space complexity and concomitant memory. A key solution has been the computation of a species–phenon relationship map, which is progressively populated by tracking down the phenon tree from top to bottom. With the resulting extended tree data structure, the internal dimensions for species boxes can then be determined and so the graphical footprint of the overarching species tree put in place.

### Evolutionary trees in *TimeScale Creator*

*TimeScale Creator* is a Java-based visualization software package which lets users draw interactive charts to explore and compare Earth-history events of any portion of geologic time. Evolutionary trees are now available for these charts (pp. 50–51 of ^81^). These trees can be displayed in columns alongside other columns such as regional lithostratigraphy, biostratigraphic zonations, isotopic curves, trends, sequences, or global and regional event time-series data. The tool to draw the new integrated species–phenon tree (pp. 52–53 of ^81^) has been included in this evolutionary-tree feature.

An evolutionary tree is inputted into our software package via a datapack containing a tab-delimited text file. Each row in this file contains text or numeric data within equivalent spreadsheet columns B–K to represent the evolutionary relationship and attributes of a time-range point. The specific format is (pp. 50–51 of  ^81^):

Data row for range

$${\rm{Label}}\,[\text{Parent} \mbox{-} \text{name}]\,({\rm{column}}\,{\rm{B}})\Rightarrow {\rm{Age}}\,({\rm{Ma}})\,({\rm{C}})\Rightarrow {\rm{Abundance}}\,({\rm{D}})\Rightarrow \text{Pop} \mbox{-} \text{up}({\rm{E}})$$

Data row for branch

$$\begin{array}{c}\text{Parent} \mbox{-} \text{name}\,({\rm{column}}\,{\rm{B}})\Rightarrow {\rm{Age}}\,({\rm{Ma}})\,({\rm{C}})\Rightarrow  \mbox{``} {\rm{branch}}\mbox{''}\,({\rm{D}})\Rightarrow \text{Child} \mbox{-} \text{name}\,({\rm{E}})\\ \Rightarrow  \mbox{``} \text{on}/\text{off}\mbox{''}({\rm{F}})\Rightarrow {\rm{BranchLabel}}\,({\rm{G}})\Rightarrow {\rm{Dashed}}/{\rm{Dotted}}\,({\rm{H}})\\ \Rightarrow \text{Pop} \mbox{-} \text{up}\,({\rm{I}})\Rightarrow {\rm{BranchColor}}\,({\rm{J}})\Rightarrow {\rm{Priority}}\,({\rm{K}})\end{array}$$

A general tree data structure is a connected network between nodes where a unique path from any ancestor to any descendant is maintained; a reverse path (from descendant to an ancestor) is not allowed. Because we are also positioning evolutionary events against standard geologic time in the evolutionary-tree column, each node in the tree corresponds to an evolutionary range (pp. 48–49 of ^81^), informing the first-appearance datum (FAD or BASE point of the range) and the last-appearance datum (LAD or TOP point of the range) for an organism. The left–right branching and spacing of descendant ranges starting at the branch point of its parent range is deduced by calculating the number of children (p. 51 of ^81^) and positioning them on the drawing canvas. Our evolutionary-tree algorithm receives only range data with point and line attributes and then generates the tree data structure determining the branch connections among parent and child ranges and hierarchies among subtrees.

The main challenge in drawing an evolutionary tree is the determination of the left–right horizontal and up–down vertical positioning of the range lines in order to avoid collisions between branches and subtrees and so generate a visually balanced tree. Our algorithm determines the width of left and right subtrees branching out from the parent ranges to recursively calculate the total width of the final tree. It draws the vertical range lines on the drawing canvas first, followed by the horizontal branch lines.

### Integrated species–phenon tree: additional programming

Drawing of the integrated tree adds a further layer of programming to that of the evolutionary tree. In particular, the width determination and positioning algorithm now needs to accommodate the breaking up of phenon range lines into parts which are then transferred across multiple rectangular species range boxes. For example, in Fig. 2i we can see that the phenon range *b* is broken into two range-line segments, the first in the species range box *Aa* and the second in *Bb*. Similarly, parts of the phenon ranges *c* and *d* are transferred from the species range box *Bb* to *Be*. This latter instance more obviously demonstrates that this breaking-up and transfer process disrupts the phenon tree structure and gives rise to disconnected subtrees within descendant species range boxes in the integrated tree. There are two new subtrees inside the species box *Be* in Fig. 2i (column c), the first one contains only the partial range line *c* and the second one is a subtree rooted at the range line *d* inside the species box *Be*. The integrated-tree algorithm needs to address the additional spacing and information transfer for these new partial ranges forming these segregated subtrees.

Programming for the integrated tree also needs to handle the possibility of phenon ranges split between several species boxes forming a successive series of ancestors and descendants. A single phenon range may be transferred to more than two species boxes depending on its first and last appearance point. The transferring of the same contents of a phenon range to its broken-up parts across multiple species boxes adds to the complications. It requires more sophisticated data structure, higher space complexity, and greater memory consumption, because there is not a fixed limit on the number of breaks in phenon range lines which may be needed. The simple counting of children per parent range under each subtree and subsequent width determination in the existing tree-drawing algorithm are not sufficient. Besides the widths of range lines and subtrees, the widths of the rectangular species range boxes need to be precisely calculated after the extended set of range lines are created. For this reason, the algorithm needs to map species–phenon relationships between an individual species range and its occupant group of phenon ranges (Table 1).

**Table 1:** Species–phenon relationship map for the integrated tree of Fig. 2i.

Relationship ID in the map	Name of species range	Size of phenon range group	Name of grouped phenon ranges	One-to-many relationship map
1-1	ancestor	1	phenon	ancestor ← phenon
2-1	*Aa*	2	*a*	*Aa* ← *a*
2-2			*b*	*Aa* ← *b*
3-1	*Bb*	4	*b*	*Bb* ← *b*
3-2			*c*	*Bb* ← *c*
3-3			*d*	*Bb* ← *d*
3-2			*f*	*Bb* ← *f*
4-1	*Be*	3	*c*	*Be* ← *c*
4-2			*d*	*Be* ← *d*
4-3			*e*	*Be* ← *e*
5-1	*Bf*	1	*f*	*Bf* ← *f*

### Integrated species–phenon tree: additional data to link phena and species

In order to link and combine the species and phenon trees, additional information needs to be provided in the datapack. However, our program minimizes this additional input by exploiting the convenient but somewhat surprising graphical fact that only one linking item is needed to accomplish this: for each phenon we need only provide the species in which its range ends. Once the top of a phenon time range is positioned in its corresponding species box, the earlier parts of its tree can be progressively transferred onto the species tree algorithmically (see previous subsection). This insight is especially handy for the user as it minimizes not only the effort needed to gather the additional data required but also the potential for errors to creep in if additional lists of mutual species–phenon memberships are compiled by other means (see § Introduction and rationale, above).

To generate an extended tree data structure the format of the input dataset of the datapack needs to be modified. The TOP range point of each phenon range line is employed to distinguish between species and phenon range lines; the tree algorithm uses this to separately create species and phenon trees. Only two entries are added to the TOP range point of an individual phenon range: (a) column G now contains the flag “phenon” and (b) column H contains the name of the species range box in which this TOP range point of the current phenon range will be positioned (see also dataset TSCEvolTree_IntTree2019Fig. 2ic.txt, § Data availability):

Data row for TOP range point

$$\begin{array}{c}{\rm{Label}}\,({\rm{column}}\,{\rm{B}})\Rightarrow {\rm{Age}}\,({\rm{Ma}})\,({\rm{C}})\Rightarrow {\rm{TOP}}\,({\rm{D}})\Rightarrow \text{Pop} \mbox{-} \text{up}({\rm{E}})\Rightarrow {\rm{blank}}\,({\rm{F}})\\ \Rightarrow  \mbox{``} {\rm{phenon}}\mbox{''}\,({\rm{G}})\Rightarrow \text{Species} \mbox{-} \text{name}\,({\rm{H}})\end{array}$$

### Integrated species–phenon tree: programming implementation

Given two side-by-side trees, the first one a species tree and the second one a phenon tree, we can establish the species–phenon mapping relationship by simply knowing which species range box will include the last appearance datum (LAD or TOP) of a phenon range. The new algorithm starts from the LAD or TOP point of a phenon range, associates this TOP point with its occupying species range line using the “phenon” flag (column H in the new format) and maps the first relationship, then continues comparing subsequent earlier (geologically past) range point ages of the phenon range line with the points of the mapped species range line. Every time it finds a younger branch connection age in the species tree than the phenon range (FAD point of the current species range greater than the phenon range points), it ends the current phenon range line under consideration by adding a new first appearance datum (FAD or BASE point). It simultaneously transfers the rest of the phenon range line to the parent species box of the current box after adding a new last appearance datum (LAD or TOP point) to the phenon range. Similarly, it continually adds new TOP and BASE points for the broken-up phenon range lines and populates the species–phenon relationship maps (Table 1) until it reaches the BASE point of the starting phenon range. This same process is applied to all the phenon range lines and, in the end, the algorithm acquires the new extended set of range lines with new range points for the phenon range lines and branch connections for further resolution of width, new symbols, and colour inheritance.

Each species range from the species tree expands horizontally to a box in the integrated tree (Fig. 2i). Each box has the exact time range (i.e., vertical extent) as on the species tree, and the branches adopted from the species tree are extended horizontally. Species boxes are basically the same species ranges with a rectangular shape and varied widths. Therefore, the boxed species tree maintains the same topology as the species tree before integration and phenon-range transfer. The species box is filled with colour that matches the colour of the inherited branch label, but with some added transparency (in the example from Fig. 2i, species box *Aa* is green but with a RGBA alpha value chosen to enable the phenon range lines, labels, and images to be visible and readable). When a colour is assigned to a branch in the datapack dataset, the ranges of its descendants inherit that colour until a new colour is assigned to a later descendant.

The choice of line styles for the range (see Fig. 2) of the species is exactly transferred to the box. In the example in Fig. 2i, all ranges except one are “frequent”, so all boxes except one have all sides with a “frequent” thickness. The exception is *Aa*, which is “frequent” from 13 to 5 Ma and “conjectured” from 5 Ma to the present. So the box is drawn with a “frequent” thickness along its base and 8 Myr up its sides from the base, and with a “conjectured” top and then 5 Myr down the sides from the top. Ranges can be assigned any of several abundance settings for any part of a range, so the equivalent species box will need to reflect this.

The width of each species box is dependent on the number of phenon ranges inside it. Using the species–phenon relationship map, the algorithm quickly resolves the group of phenon ranges to be drawn inside each species box. It takes consideration of the width of both the range label and image for each phenon range. It is also possible that there may exist a species box without phenon ranges; these are given a minimum width to handle such exceptions.

Each instance of the phenon range retains its core features. When phenon splits are required, all the features of the phenon ranges are duplicated and attached to each broken-up portion, e.g., the thumbnail image and pop-ups. However, the line style of the phenon ranges strongly follows the parent phenon tree because line styles are directly associated with the abundance details (line styles) for the phenon range. Visual distinction between species and phena is accomplished by employing different settings for the “default” line styles (frequent vs. common, respectively). The species range box boundaries are therefore thicker than the phenon range line (Fig. 2i, column c).

In order to make it easier to appreciate the context of phenon range lines, especially to distinguish between their ranges and their parts broken between species boxes, guide symbols are added to the extremities of each of their parts (Fig. 2ii).

### Integrated species–phenon tree: disconnected trees and phenon ranges

Our integrated-tree tool can manage multiple phenon trees (Fig. 2iii: original coloured tree, with additional phenon tree *h*–*i* in black). If the datapack contains more than one mutually unconnected tree, the preexisting evolutionary-tree algorithm has been retained to draw all trees within the same *TimeScale Creator* column, positioning them side by side based on “First occurrence”, “Last occurrence” or “Alphabetic order” (p. 49 of  ^81^).

Our integrated tree can also handle disconnected phenon ranges: not all of the phenon ranges need to be part of a tree structure (Fig. 2iii: additional phenon *g* in black). The algorithm orders the phenon ranges using the “First Occurrence” by default. But three options, (a) First Occurrence, (b) Last Occurrence, and (c) Alphabetic Order, are provided to the user from the settings window of the software. Ordering of the phenon ranges inside the species box can thus be freed from the topology of the unified phenon tree.

### Integrated species–phenon tree: short phenon labels

The integrated tree allows alternative labels for phena, usually to conserve space. Another application for this could be to apply a particular nomenclatural format for phenon labels. For instance, if a study conferred scientific names on phena, one could opt for phena to be given only a single species epithet (*species*, of *Genus species*), and so distinguish them from binomina (*Genus species*) of their enveloping species boxes. This feature is implemented by placing “|” within the phenon range name in the dataset, in which case the text after “|” is excluded; if the name does not contain “|”, then the full name is used. (This option was effected in the back-end database of the case study via the table SpeciesGroupName, in which the original assignations of scientific names are rearranged to begin with the most trivial name — in order to provide an index invariant to later nomenclatural revisions; see links to details of the database in Table 2 and Fig. 8 of  ^54^. For example, to take morphospecies *Acarinina bullbrooki*, featured in Fig. 3d, its original assignation, “*Globorotalia bullbrooki* Bolli, 1957”, rearranges to “*bullbrooki* Bolli, 1957; *Globorotalia*”. If then the phenon range name in the dataset is given as “*bullbrooki* | Bolli, 1957; *Globorotalia*”, the phenon range label displayed on the integrated tree will be reduced to “*bullbrooki*”). If the user’s key concern is to more distinctly distinguish visually between species and phenon labels, the fonts for each can be separately manipulated (*TimeScale Creator* settings: Choose Time Interval, select datapack column, Fonts).

## Data Availability

The *TimeScale Creator* datapacks introduced herein for the integrated species–phenon tree from the case study of Cenozoic macroperforate planktonic foraminifera are available at Australian National University Data Commons collection anudc:5981, 10.25911/5db66faba683b^82^. Datapacks Coloured by ecogroup With labels: TSCEvolTree_Aze&2011_CorrJul2018_ISPEco.dpk Without labels: TSCEvolTree_Aze&2011_CorrJul2018_ISPEcoNoLbl.dpk. Coloured by morphogroup With labels: TSCEvolTree_Aze&2011_CorrJul2018_ISPMph.dpk Without labels: TSCEvolTree_Aze&2011_CorrJul2018_ISPMphNoLbl.dpk. Settings: TSCEvolTree_Aze&2011_CorrJul2018_ISP_4dpks.tsc. Note that these datapacks do not include the images of foraminifera featured in Fig. 3, because authoritative images are not yet available for these datasets. These datapacks are also freely available from the *TimeScale Creator* website https://timescalecreator.org/datapack/datapack.php Other Supplementary Information for this article is available at Australian National University Data Commons collection anudc:5982, 10.25911/5db66fd8c5127^83^. *TimeScale Creator* datapacks for Fig. 2i Datasets TSCEvolTree_IntTree2019Fig. 2ia,b.txt TSCEvolTree_IntTree2019Fig. 2ic.txt Datapacks TSCEvolTree_IntTree2019Fig. 2ia,b.dpk TSCEvolTree_IntTree2019Fig. 2ic.dpk Settings TSCEvolTree_IntTree2019Fig. 2ia,b.tsc. TSCEvolTree_IntTree2019Fig. 2ic.tsc *TimeScale Creator* datapacks for Fig. 2iii Datasets TSCEvolTree_IntTree2019Fig. 2iiia,b.txt TSCEvolTree_IntTree2019Fig. 2iiic.txt Datapacks TSCEvolTree_IntTree2019Fig. 2iiia,b.dpk TSCEvolTree_IntTree2019Fig. 2iiic.dpk Settings TSCEvolTree_IntTree2019Fig. 2iiia,b.tsc. TSCEvolTree_IntTree2019Fig. 2iiic.tsc “Functional Specification for the Evolutionary Trees function, providing an option for an Integrated Species–Phenon Tree, 27 August 2014”: Functional Specification _ Integrated Species – Phenon Tree.docx. *TimeScale Creator* datapacks, the relational database, and related files for the previously published lineage and morphospecies trees from the case study are also available at Australian National University Data Commons collections (for details see^54^). These datapacks are also freely available from the *TimeScale Creator* website.

## Code availability

The source-code files for the Integrated Species–Phenon Tree feature are available at https://github.com/brishtiteveja/TSCreator_Integrated_Evolutionary_Tree and at

Australian National University Data Commons collection anudc:5980, 10.25911/5db66f7bc1935^84^. These repositories contain the source-code files constituting the core programming for the Integrated Species–Phenon Tree feature within the Evolutionary Tree column of the *TimeScale Creator* software platform.

RangeColumn.java: includes detailed data structures for evolutionary range points and ranges, and tree-drawing utility functions.

ImageGenerator.java: draws the evolutionary tree data column and generates the final chart.

Main.java: includes the main function which is the entry point to the software and spawns the Java process on a Java Virtual Machine.

Readme.txt.

The *TimeScale Creator* software platform is freely available at https://timescalecreator.org/download/download.php, from where it can be downloaded as a Java archive (jar) file or Windows executable (exe) file. Instructions to install *TimeScale Creator* and load datapacks are given in Instructions_for_TSCreator_datapacks.pdf, included in anudc:5980.
